# Bacterioruberin: Biosynthesis, Antioxidant Activity, and Therapeutic Applications in Cancer and Immune Pathologies

**DOI:** 10.3390/md22040167

**Published:** 2024-04-09

**Authors:** Micaela Giani, Carmen Pire, Rosa María Martínez-Espinosa

**Affiliations:** 1 Multidisciplinary Institute for Environmental Studies “Ramón Margalef”, University of Alicante, Ap. 99, E-03080 Alicante, Spain; micaela.giani@ua.es (M.G.); carmen.pire@ua.es (C.P.); 2 Biochemistry and Molecular Biology and Edaphology and Agricultural Chemistry Department, Faculty of Sciences, University of Alicante, Ap. 99, E-03080 Alicante, Spain

**Keywords:** bacterioruberin, haloarchaea, C_50_ carotenoids, antioxidant, immunomodulation, antitumoral

## Abstract

Halophilic archaea, also termed haloarchaea, are a group of moderate and extreme halophilic microorganisms that constitute the major microbial populations in hypersaline environments. In these ecosystems, mainly aquatic, haloarchaea are constantly exposed to ionic and oxidative stress due to saturated salt concentrations and high incidences of UV radiation (mainly in summer). To survive under these harsh conditions, haloarchaea have developed molecular adaptations including hyperpigmentation. Regarding pigmentation, haloarchaeal species mainly synthesise the rare C_50_ carotenoid called bacterioruberin (BR) and its derivatives, monoanhydrobacterioruberin and bisanhydrobacterioruberin. Due to their colours and extraordinary antioxidant properties, BR and its derivatives have been the aim of research in several research groups all over the world during the last decade. This review aims to summarise the most relevant characteristics of BR and its derivatives as well as describe their reported antitumoral, immunomodulatory, and antioxidant biological activities. Based on their biological activities, these carotenoids can be considered promising natural biomolecules that could be used as tools to design new strategies and/or pharmaceutical formulas to fight against cancer, promote immunomodulation, or preserve skin health, among other potential uses.

## 1. Introduction

Most moderate and extreme halophilic microorganisms require salt concentrations above 1 M to be alive and metabolically active, although several species can endure a spectrum of saline conditions, ranging from low-salt to salt saturation concentrations (around 4 M) [1]. Based on the ionic strength required by the cells for stability and metabolic activity, microbial halophilic microorganisms are mainly classified into the following categories: slight halophiles (0.34 to 0.85 M), moderate halophiles (0.85 to 3.4 M), and extreme halophiles (3.4 M to saturation point) [2].

Studies based on microbial ecology and microbial biodiversity in salty aquatic environments confirmed that halophilic archaea species (also termed “haloarchaea”) constitute the predominant populations, especially at high salt concentrations (2–4 M), apart from some bacterial genera like *Salinibacter* [3]. At significantly high salt concentrations, halophilic archaea belonging to the families *Halobacteriaceae* and *Haloferacaceae* (phylum Euryarchaeota within the Archaea domain) are the most abundant populations [4]. These halophilic archaea are widely distributed in saline environments, such as marshes or brackish ponds, which serve as sources of NaCl for human consumption [5]. Halophilic archaea primarily thrive in aerobic conditions, although certain species exhibit the capability to grow under microaerobic or even anaerobic conditions, utilising nitrate and/or nitrite as the final electron acceptors through denitrification processes [6]. Additionally, a noteworthy characteristic of these archaea is that most of them typically show a distinctive red/orange pigmentation, thus providing these kinds of colours to the salted ponds where they live all together with pigmented yeast and microalgae like *Dunaliella salina* [7]. These pigmented halophilic archaea have mainly been isolated from water column and sediment sampling at hypersaline environments such as hypersaline lakes such as the Great Salt Lake in Utah, USA; salt marshes; coastal wetlands; or solar salterns like those located in the south and southeast of Spain (Alicante, Murcia, or Huelva) [5].

Haloarchaea have attracted global scientific attention due to their unique features related to the molecular machinery of nitrogen, biodegradable polymers, and carotenoid metabolism; their easy manipulation; their reduced space requirements for cultivation compared to other organisms like microalgae or yeast from which highly marketed compounds can be obtained; and their capacity to produce a wide array of biomolecules and metabolites with potential biotechnological applications compared to plants, bacteria, fungi, or eukaryotic algae [8,9]. Their remarkable resilience and functionality even in the face of challenging environmental conditions, including high salinity, intense ultraviolet (UV) radiation, elevated ion concentrations, and extreme temperatures and pH, make them good model organisms to be used as cell factories for different purposes compared to their bacterial counterparts [10,11,12].

Among the biomolecules or metabolites of biotechnological interest synthesised by haloarchaea, small proteins and peptides, enzyme bioplastics, carotenoids, and nanoparticles can be highlighted. The carotenoids found in haloarchaea have garnered significant attention in various industries due to their versatility, serving as antioxidants, anticancer agents, antimicrobials, anti-inflammatory compounds, and food colourants, thus offering numerous biotechnological and biomedical applications [13,14,15]. Bacterioruberin (BR), the main carotenoid pigment synthesised by halophilic archaea, has demonstrated notable biological activity, particularly in antioxidant properties [14,16,17,18,19,20]. Understanding its biosynthesis pathways and structural features can provide insights into its potential therapeutic applications. This review focuses on the multifaceted biological activity of BR obtained from haloarchaea due to their better characterisation and the abundance of literature available on these microorganisms. BR, known for its characteristic red colouration, exhibits remarkable antioxidant properties owing to its ability to scavenge reactive oxygen species (ROS) and mitigate oxidative stress [21,22,23]. Additionally, recent studies have unveiled its potential immunomodulatory effects, suggesting a role in modulating immune responses and inflammation [24,25]. Therefore, advances in the comprehension of the pharmacological relevance of bacterioruberin hold promise for developing novel therapeutic interventions targeting oxidative-stress-related disorders, immune system dysregulation, and potentially other pathological conditions.

## 2. Characteristics of Haloarchaeal Carotenoids and Their Biological Roles

### 2.1. Chemical Composition and Structure

The literature concerning carotenoids synthesised by haloarchaea is still scarce compared to the literature available on carotenoids from other living beings, such as plants, algae yeast, and fungi [26,27,28,29,30,31,32]. The first reported studies on carotenoids isolated from haloarchaea date back to the 1970s, using *Halobacterium cutirubrum* as a model microorganism [33,34]. At that time, it was confirmed that the major carotenoid produced by the cells is the rare C_50_ BR followed by monoanhydrobacterioruberin (MABR) at the expense of lycopene and bisanhydrobacterioruberin (BABR); both MABR and BABR are precursors of BR [33,34]. Other carotenoids have been identified in haloarchaeal carotenoid extracts but at lower percentages, including β-carotene, lycopene, and some xanthophylls [35,36]. Structurally, BR is very unique among carotenoids (being considered a “rare C_50_ carotenoid” by several authors), consisting of a primary conjugated isoprenoid chain that contains 13 conjugated double bonds and four hydroxyl groups arising from the terminal ends. Table 1 displays the chemical structure of BR and its precursors (Table 1) [37].

Although BR is produced almost exclusively by haloarchaea, some studies confirmed that few bacterial species showing extreme phenotypes like the Antarctic psychrotrophic bacterium *Micrococcus roseus* and *Arthrobacter* species are also able to synthesise it [38,39,40].

### 2.2. Carotenogenesis and Biological Role of Carotenoids in Haloarchaeal Cells

The synthesis of carotenoids (also termed “carotenogenesis”) and its regulation have not been studied as deeply in haloarchaea [26,41,42,43,44] as they have in bacteria, yeasts, or plants [27,45,46,47,48,49,50,51,52]. In brief, haloarchaea use the mevalonate pathway to produce the carotenoid precursor isopentenyl pyrophosphate. Then, it is converted into trans-phytoene, which leads to ζ-carotene which is further converted to neurosporene. Neurosporene is transformed into lycopene, from which most carotenoids derive, including β-carotene with Υ-carotene as an intermediate compound. Bacterioruberin is also synthesised from lycopene [26,41]. The main reaction involved is the addition of C_5_ isoprene units to each end of the lycopene structure. However, in contrast to other secondary metabolites, the enzymes related to carotenogenesis are not always encoded within the same gene cluster [41] and some paralogs do not serve functional roles, as recently reported by Mishra and collaborators [53]. This circumstance poses a challenge in accurately identifying complete carotenogenesis pathways in each strain. Although other xanthophylls, including cantaxanthin and astaxanthin, have been detected in carotenoid extracts obtained from haloarchaeal cells [36,54], the genes coding for enzymes involved in their synthesis have not been identified in haloarchaeal genomes [41]. More recently, Serrano and collaborators reported that β-carotene could be converted to canthaxanthin by the action of a β-carotene ketolase protein. Through genome analysis, the authors identified a crtO candidate in one of the six circular plasmids in *Haloterrigena turkmenica*, which suggests that this species can produce canthaxanthin from β-carotene [26]. CrtO ketolases are structurally related to CrtI phytoene desaturases [55,56]. In consequence, haloarchaea without CrtO-like genes could still be producing canthaxanthin expressing one of their sometimes multiple CrtI genes [26]. Alternative unidentified pathways for the synthesis of these carotenoids could be a possible explanation.

Among all the carotenoids mentioned, BR is the most abundant natural carotenoid produced by haloarchaea. It is responsible for the intense pink colour since it is located in the cell membrane of the haloarchaea that synthesise it [57]. Its long hydrocarbon chain makes it able to fit in between the glycerolipids forming the bilayer, with the hydroxyl group facing outwards and inwards. Haloarchaeal pigments play a pivotal role in membrane stability, acting as a protection mechanism against the harsh conditions usually present in the natural environment of these extremophilic microorganisms, such as high oxidative and osmotic stress and elevated radiation [21,39,57,58]. BR protects cells from oxidative damage by acting as an antioxidant thanks to the electron transport between the pairs of conjugated double bonds. Since bacterioruberin presents a longer hydrocarbon chain and a higher number of conjugated double bonds than other carotenoids, such as β-carotene (C_40_ carotenoid, nine conjugated double bonds), it has extraordinary scavenging activity [14,16,18,59,60,61,62].

In addition, the presence of bacterioruberin in the cell membrane increases the rigidity and decreases water permeability, while allowing oxygen to enter inside the cell [63]. Thus, cells are capable of modulating membrane rigidity in a wide range of temperatures as well as in a concrete range of salinity conditions.

Finally, BR contributes to maintaining the structural stability of rhodopsin complexes. More specifically, some studies have demonstrated that it is associated with archaerhodopsin, which is a complex formed by a retinal protein and a carotenoid, identified in some haloarchaeal species, including *Halobacterium salinarum* [64,65,66].

## 3. Antioxidant Properties of Bacterioruberin and Its Precursors

Advances in the knowledge of the biological activities of BR have revealed that it could be of interest in several industrial and biomedical sectors due to its high antioxidant activity [14,15,25,59,60,61,67,68]. Based on the chemical composition and structure of BR, it was initially assumed that this natural carotenoid has strong antioxidant properties as it was quantified later compared to one of the most marketed carotenoids, β-carotene [14,16,18,59,60,61,69,70]. This is probably the biological activity that is better characterised in the case of BR (Table 2).

A recent study has reported how the modification of the nutritional conditions of *Haloferax mediterranei* during growth can lead to changes in the carotenoid extract composition and, in consequence, its properties and antioxidant capacity [14]. The combination of 2.5% (*w*/*v*) glucose with 12.5% (*w*/*v*) salinity led to a carotenoid extract with an IC_50_ value of 0.03 µg/mL in the ABTS (2,2′-Azinobis-(3-Ethylbenzthiazolin-6-Sulfonic Acid) assay. This value was lower than the one obtained for ascorbic acid [14], coinciding with other reported results and confirming the remarkable antioxidant properties of these complex extracts [19,20]. Other researchers have explored the activity of carotenoid extracts of *Haloarcula hispanica* and *Halobacterium salinarum*, observing that they could scavenge DPPH (2,2-diphenyl-1-picrylhydrazyl) (2.05 µg/mL and 8.9 µg/mL) and ABTS radicals (3.89 µg/mL and low activity for *H. salinarum*) and reduce ferrocyanide and chelate copper, but not scavenge NO radicals or chelate iron [60]. Carotenoid extracts from *Halococcus morrhuae* and *H. salinarum* presented IC_50_ values of 0.85 µg/mL and 0.84 µg/mL for the ABTS assay [18]. *Haloferax* sp. ME16, a haloarchaeal strain isolated from Algerian salt lakes, displayed significantly higher antioxidant power than ascorbic acid in both the DPPH and ABTS assays [69]. Recently, carotenoid extracts from *Halorhabdus utahensis* exerted their scavenging power in a set of different antioxidant assays (DPPH, FRAP, and Superoxide Scavenging Activity assays), confirming that haloarchaeal carotenoids have a broad range of modes of action against oxidants [14,17]. A similar approach (ABTS, FRAP, and DPPH) was chosen to evaluate the antioxidant activity of *Natronoccoccus* sp. TC6 and *Halorubrum tebenquichense* carotenoid extracts. Although the extracts were efficient in all tests, they showed a dominating capacity of hydrogen and single electron transfer [68]. In summary, there are notable differences in the antioxidant activity of carotenoid extracts from different haloarchaeal species. There are different possible explanations for the variability in the results. It is possible that halophilic archaea could synthesise antioxidant compounds of different natures and polarities that contribute independently to the total antioxidant activity of the extracts, and this might be dependent on the haloarchaeal species but also influenced by the growth conditions. However, if this is the case, the concentration of those compounds should be significantly low because they cannot be easily detected by standard chromatographic-based approaches, at least those used in the case of *Haloferax mediterranei* extracts. One of the greatest difficulties when comparing current studies is that although they all agree that the all-trans-BR isomer is the most abundant, not all of them include the percentages of the different components of the extract, which in turn could be one of the factors influencing the antioxidant activity. Aside from that, there are experimental differences that might also influence the results obtained in the antioxidant tests, such as the organic solvent used for the extraction of carotenoids.

In addition, BR and intracellular KCl in *Halobacterium salinarum* and *Halobacterium* sp. strain NRC1 act as a protective mechanism against oxidative DNA damage induced by UV radiation. Therefore, this carotenoid might have potential applications in medicine and cosmeceuticals focused on the mitigation of DNA damage and the preservation of cellular integrity [71,72]. Understanding these protective mechanisms could inspire the development of novel therapies and skincare products targeting oxidative-stress-induced DNA damage, offering promising applications in healthcare and cosmetic industries.

**Table 2:** Antioxidant activity of BR with potential applications in food, cosmetics, and pharmacy.

Species	Aim of the Research	Reference
*Halorubrum ruber*	Optimisation of BR production and analysis of the effect of its antioxidant activity on the survival rate of *Caenorhabditis elegans* under oxidative stress conditions	[23]
*Halorubrum ezzemoulense*	Description of the effects of BR on the thermal and oxidative stabilities of fish oil	[22]
*Haloarcula japonica* *Haloarcula salaria* *Halococcus morrhuae* *Halobacterium salinarium* *Haloferax alexandrinus* GUSF-1 *Haloferax* sp. ME16 *Halogeometricum* sp. ME3 *Haloarcula* sp. BT9 *Halorhabdus utahensis* *Halorubrum chaoviator* *Halorubrum lipolyticum* *Halorubrum sodomense* *Halorubrum* sp. BS2 *Halorubrum tebenquichense* SU10 *Haloterrigena turkmenica* *Natronoccoccus* sp. TC6	Isolation and characterisation of the total carotenoid extract and antioxidant activity quantification	[16,17,18,19,68,69,70,73]

## 4. Immunomodulatory/Anti-Inflammatory Activities of BR Collectively with Antioxidant Activity

In connection with the antioxidant activity, recent studies have described the anti-inflammatory activities and immunomodulatory benefits of BR on human commercial cell lines. For example, *Haloarcula* sp. isolated from Odiel Saltworks (south of Spain) was used as the source of a carotenoid extract which is rich in BR and C_18_ fatty acids. This extract showed potent antioxidant capacity using the ABTS assay. This study further demonstrates that pretreatment with this carotenoid-rich extract of lipopolysaccharide (LPS)-stimulated macrophages resulted in a reduction in ROS production, a decrease in the pro-inflammatory cytokines TNF-α and IL-6 levels, and an upregulation of the factor Nrf2 and its target gene heme oxygenase-1 (HO-1), supporting the potential of the carotenoid extract as a therapeutic agent in the treatment of oxidative-stress-related inflammatory diseases [24].

Similarly, another study carried out with BR from *Halorubrum tebenquichense* suggested that the carotenoid in combination with dexamethasone (Dex) in ultra-small macrophage-targeted nanoparticles could act as a potential intestinal repairing agent [25]. The ultra-small structures in which BR and dexamethasone were embedded were extensively captured by macrophages and Caco-2 cells and displayed high anti-inflammatory and antioxidant activities on a gut inflammation model made of Caco-2 cells and lipopolysaccharide-stimulated THP-1-derived macrophages, reducing 65% and 55% of TNF-α and IL-8 release, respectively, and 60% of reactive oxygen species production. The ultra-small structures also reversed the morphological changes induced by inflammation and increased the transepithelial electrical resistance, partly reconstituting the barrier function. The main conclusion was that this nanostructure containing BR and Dex deserves further exploration as an intestinal-barrier-repairing agent [25].

In summary, while the evidence published until now suggests the therapeutic potential of bacterioruberin (BR) in mitigating oxidative-stress-related inflammatory diseases and promoting intestinal repair, it is important to note that the current body of research is limited, and further studies are needed to establish robust conclusions. These initial studies underscore BR’s antioxidant capacity and its potential anti-inflammatory and immunomodulatory effects. The utilisation of BR-rich extracts or BR in combination with drugs within innovative nanostructures shows promise for addressing several pathologies. Nonetheless, the existing findings highlight the need to further research and develop BR-based formulations, elucidate underlying mechanisms, and assess the safety and efficacy of BR in clinical settings.

## 5. Antitumoral Properties of Bacterioruberin and Its Precursors

The effect of BR on tumoral cells has been recently explored. Thus, carotenoids (0.2–1.5 μM) from a haloarchaeal strain (M8) could reduce up to 50% hepatoma cell line (HepG2) viability in a concentration-dependent way. In addition, hepatoma cells treated with haloarchaeal carotenoids were less sensitive to oxidative stress generated by H_2_O_2_, thus exerting a protective effect [59]. The antiproliferative effect on hepatoma cells was also reported for extracts obtained from *Halogeometricum limi* and *Haloplanus vescus* [61]. These extracts also presented antihemolytic activities against H_2_O_2_-induced hemolysis in mouse erythrocytes [61]. The anticancer effect of *Natrialba* sp. M6 carotenoid extract was reported again for hepatoma cells (HepG2) as well as for other types of cancer cell lines, including Caco-2 (colon cancer), MCF-7 (breast cancer), and HeLa (cervical cancer) [74]. In the case of MCF-7 commercial cell lines, a real-time PCR technique was used to monitor the expression of genes specific for apoptosis, in the presence or absence of BR-rich carotenoid extract. Both early and late apoptosis were increased significantly by about 10% and 39%, respectively, due to the upregulation of CASP3, CASP8, and BAX gene expression in the MCF-7 cell line. In contrast, the expression of the genes MKI67 and SOX2 was significantly downregulated in the treated MCF-7 cell line. The results of this study showed that the carotenoid extract isolated from *Haloarcula* sp. A15 could be a good candidate for the production of high-added-value bacterioruberin due to its possible anticancer properties [75]. The antiproliferative effect on breast cancer cell lines has been explored in other studies [15,75]. In particular, *Haloferax mediterranei* carotenoid extracts reduced cell adhesion, viability, diameter, and cell concentrations in cell lines representative of the four well-defined subtypes of breast cancer (Luminal A, Luminal B, HER2-enriched, and triple-negative) [15].

In conclusion, the observed antiproliferative effects of BR-rich extracts from various haloarchaeal strains, notably on hepatoma and breast cancer cell lines, suggest its potential as a valuable candidate for novel anticancer therapies. However, to translate these findings into clinically relevant interventions, further investigations are necessary to elucidate the underlying molecular mechanisms driving BR’s anticancer properties. Additionally, comprehensive studies are needed to assess potential interactions between BR and current anticancer drugs, ensuring their compatibility and optimizing therapeutic outcomes. It is important to acknowledge the limitations associated with the use of carotenoids, including challenges in identifying optimal doses and potential variations in bioavailability. Moreover, while current preliminary studies may focus on treatment perspectives, clinical investigations with other carotenoids often adopt a preventive approach, which limits the accuracy of direct comparisons. Addressing these complexities will be essential for advancing our understanding of BR’s therapeutic potential and developing effective strategies for cancer management.

## 6. Other Biological Activities of Interest for Biomedical and Pharmaceutical Applications

Aside from their antitumor activity, haloarchaeal carotenoids could have an impact on diabetes and obesity treatments. *Haloferax mediterranei* carotenoid extracts are capable of inhibiting α-glucosidase, α-amylase, and lipase enzymes which are involved in carbohydrate and lipid metabolism. The inhibition of these enzymes is the target of several drugs used to reduce blood glucose and lipid absorption, respectively [14]. Carotenoids from *Halorhabdus utahensis* reached 90% hyaluronidase inhibition with 1.5 μg, demonstrating great potential for applications in the skin care sector [17].

*Halobacterium salinarum* and *Haloarcula hispanica* carotenoid extracts can inhibit COX-2, acetylcholinesterase, and tyrosinase enzymes and, therefore, they could have potential applications as a treatment for inflammatory, neurological, and dermatological diseases [60]. In addition, haloarchaeal carotenoids exert antimicrobial activity against a wide range of species. For example, bacterioruberin from *Halorubrum tebenquichense* inhibited Staphylococcus aureus growth and biofilm formation [76]. Table 3 summaries more examples in which other biological activities have been described for BR.

Finally, although synthetic colourants have been extensively used for numerous years in the cosmetics industry, their detrimental impacts on both the environment and health should not be disregarded. It is key to explore natural alternatives, with a particular focus on microbial colourants, to increase safety and reduce potential side effects [77,78,79,80]. Currently, there are multiple companies whose objective is to produce cosmetic and cosmeceutical products that are visually attractive as well as respectful of the environment and human health using ingredients of natural origin. In parallel, natural carotenoids are of interest and currently highly demanded by textile industries. Carotenoids from biological sources are increasingly used as ingredients in these kinds of formulations, due to both their colouring and biological properties [81]. β-carotene is one of the most frequently used carotenoids in this field and it can be obtained from the halophilic microalgae *Dunaliella salina* [82]. Other carotenoids that have been included in currently marketed cosmetic products are astaxanthin (*Haematococcus pluvialis*) and fucoxanthin (*Phaeodactylum tricornutum*) [81].

BR emerges as a promising candidate due to its intense pink-red hue and notable biological properties. As discussed earlier, BR possesses antioxidant, anti-inflammatory, and immunomodulatory characteristics, making it not only aesthetically appealing but also potentially beneficial for skin health. Its natural origin from halophilic archaea aligns with the growing consumer demand for eco-conscious and sustainable products. Despite its promising biological properties, its suitability for topical use on the skin is yet to be addressed. Investigating bacterioruberin’s interactions with skin cells, its ability to penetrate the epidermal barrier, and its potential benefits in addressing dermatological conditions or enhancing cosmetic formulations would be invaluable.

BR’s potential as a colouring agent also remains relatively unexplored. Given its intense pink-red hue, bacterioruberin holds promise as a natural and vibrant alternative to synthetic colorants in various applications. However, there is a notable gap in research regarding its efficacy, stability, and safety as a colouring agent, particularly in food, cosmetics, and textile industries. Understanding bacterioruberin’s colour stability under different processing conditions, its compatibility with various matrices, and its potential allergenicity or toxicity is crucial for its widespread adoption as a colouring agent. Furthermore, exploring methods for extracting and purifying bacterioruberin on an industrial scale is essential for its commercial viability. By bridging these knowledge gaps, BR could be an alternative natural and sustainable colorant with diverse applications across industries.

## 7. Conclusions

Haloarchaeal carotenoids could have a diverse range of applications across the fields of biomedicine, food processing and conservation, pharmaceuticals, and textiles. These naturally occurring pigments have emerged as promising alternatives to synthetic colourants, addressing the growing demand for natural food colouring options worldwide as well as natural colourants as part of the formulations in cosmetics and pharmacology. Because of the antioxidant, anti-inflammatory, immunomodulatory, and antitumoral activities of BR, this C_50_ carotenoid offers new approaches and strategies for defining new drug formulations or drug immobilisation techniques as part of the treatments of pathologies related to the immune system and cancer, among others. By promoting research on haloarchaeal pigments, it is possible to uncover novel applications for these promising C_50_ carotenoids. Furthermore, the cultivation of haloarchaea and carotenoid extraction are more straightforward compared to other living beings, making them attractive subjects for the research and development of sustainable processes aiming at the production of natural pigments with a wider spectrum of applications, all following circular economy-based processes.

## Figures and Tables

**Table 1:** Structures and common and scientific names of bacterioruberin (BR) and its precursors.

Common Name	Molecular Formula	Chemical Structure (Stereoisomers)
Bacterioruberin	C_50_H_76_O_4_	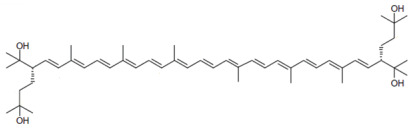 (2*S*,2′*S*)-2,2′-bis(3-hydroxy-3-methylbutyl)-3,4,3′,4tetrahydro-γ,γ-carotene-1,1′-diol
Monoanhydrobacterioruberin	C_50_H_74_O_3_	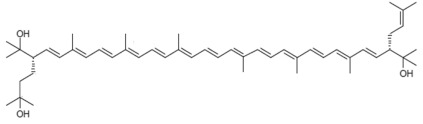 (3*S*,4*E*,6*E*,8*E*,10*E*,12*E*,14*E*,16*E*,18*E*,20*E*,22*E*,24*E*,26*E*,28*E*,30*S*)-30-(2-hydroxypropan-2-yl)-2,6,10,14,19,23,27,33-octamethyl-3-(3-methylbut-2-en-1-yl)tetratriaconta-4,6,8,10,12,14,16,18,20,22,24,26,28-tridecaene-2,33-diol
Bisanhydrobacterioruberin	C_50_H_72_O_2_	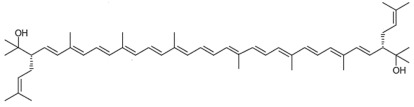 (3*S*,4*E*,6*E*,8*E*,10*E*,12*E*,14*E*,16*E*,18*E*,20*E*,22*E*,24*E*,26*E*,28*E*,30*S*)-2,6,10,14,19,23,27,31-octamethyl-3,30-bis(3-methylbut-2-en-1-yl)dotriaconta-4,6,8,10,12,14,16,18,20,22,24,26,28-tridecaene-2,31-diol

**Table 3:** Other biological activities of BR and its precursors with potential uses in food, cosmetics, medicine, and pharmacy.

Species	Aim of the Research	Reference
*Natronoccoccus* sp. *Halorubrum tebenquichense*	Matrix metallopeptidase 9 (MMP-9) inhibition activities	[68]
*Haloferax mediterranei*	Characterisation of antiglycaemic and antilipidemic activities	[14]
*Haloferax* sp. ME16 *Halogeometricum* sp. ME3*Haloarcula* sp. BT9	Characterisation of the antibacterial activity of BR-rich extracts	[69]
*Halorubrum* sp. BS2	Isolation and characterization of total carotenoid extracts, and antibacterial activity quantification	[70]
*Natrialba* sp. M6	Isolation and characterisation of total carotenoid extracts, and antiviral activity quantification	[74]
*Haloferax volcanii*	Bioactive properties of BR on sperm cells (mainly in connection with antioxidant properties)	[62]
*Halogeometricum rufum* *Halogeometricum limi* *Haladaptatus litoreus* *Haloplanus vescus* *Halopelagius inordinatus* *Halogranum rubrum* *Haloferax volcanii*	Antihaemolytic activity apart from antitumoral and antioxidant activities	[61]
